# Characteristics of clinical trials associated with appeal and return on investment to participants: a review and framework

**DOI:** 10.1093/oncolo/oyaf313

**Published:** 2025-09-27

**Authors:** Maria J Fernandez Turizo, Maria A Velez, Beth Glenn, Amy L Cummings, Barbara Segarra-Vazquez, Sarah Gorbatov, Seung J Park, Collin Shen, Jackson P Lind-Lebuffe, Joseph M Unger, Edward B Garon

**Affiliations:** Department of Medicine, Beth Israel Deaconess Medical Center , Deaconess Building, Suite 306, One Deaconess Road, MA 02215, United States; Department of Medicine, Division of Hematology and Oncology, University of California Los Angeles, 2020 Santa Monica, Suite 600, Santa Monica, CA 90404, United States; Department of Health Policy and Management in the UCLA Fielding School of Public Health, University of California, Los Angeles, 31-269 Center for Health Sciences, Los Angeles, CA 90095, United States; Department of Medicine, Division of Hematology and Oncology, University of California Los Angeles, 2020 Santa Monica, Suite 600, Santa Monica, CA 90404, United States; Department of Graduate Programs, School of Health Professions, Medical Sciences Campus, University of Puerto Rico, San Juan, Puerto Rico 00921, United States; Department of Medicine, Division of Hematology and Oncology, University of California Los Angeles, 2020 Santa Monica, Suite 600, Santa Monica, CA 90404, United States; Department of Medicine, Division of Hematology and Oncology, University of California Los Angeles, 2020 Santa Monica, Suite 600, Santa Monica, CA 90404, United States; Department of Medicine, Division of Hematology and Oncology, University of California Los Angeles, 2020 Santa Monica, Suite 600, Santa Monica, CA 90404, United States; Department of Medicine, Division of Hematology and Oncology, University of California Los Angeles, 2020 Santa Monica, Suite 600, Santa Monica, CA 90404, United States; Fred Hutchinson Cancer Center, 1100 Fairview Ave N, Seattle, WA 98109, United States; Fred Hutchinson Cancer Center, 1100 Fairview Ave N, Seattle, WA 98109, United States

**Keywords:** oncology clinical trials, clinical trial design, Return on Investment

## Abstract

Despite decades of investment in clinical research infrastructure, patient participation in clinical trials remains strikingly low. In the United States, fewer than 1 in 10 adults report ever being invited to participate in a clinical trial, and among those, less than half ultimately enroll. In oncology, across all adult cancer patients, only about 8% enroll in a clinical trial, regardless of eligibility or trial availability. Active engagement of their patients in cancer clinical trials substantially enhances scientific knowledge, and patient participation is required to obtain approval for novel therapeutics. Analyses focusing on evaluating whether clinical trial participation improves survival for participants have yielded conflicting results. Yet, there is limited data or metrics to assess the specific attributes of oncology trials that hold greater appeal or return on investment to participants, limiting researchers’ ability to determine if these factors vary across different populations. Our group demonstrated that patients with limited English proficiency were unlikely to participate in studies not sponsored by industry as compared to industry-sponsored studies. If trial participation for specific populations can differ by sponsor type, it could also differ by the trial’s appeal or return on investment. While the underrepresentation of racial and ethnic minority groups in trials is attributed to multiple factors, it is possible that patients from these groups are less likely to be offered participation in studies with higher appeal or return on investment, due to systemic biases, disparities in recruitment practices, and/or lack of access. In this manuscript, we propose a systematic framework for evaluating attributes of interventional oncology clinical trials, with an emphasis on the study’s purpose, experimental design, and investigational agent. This framework aims to pinpoint characteristics that may enhance trials’ appeal or return on investment to participants and could lay a foundation for future research. This will allow investigators to assess the return on investment of appeal of studies offered across different patient populations.

Implications for practice Current evidence is conflicting on whether participation in a clinical trial increases patient survival. This review proposes both a retrospective and a prospective framework for evaluating appeal and return on investment to participants in interventional oncology studies. In other words, the framework aims to capture what patients with cancer may perceive as meaningful benefits when deciding whether to enroll in a trial. In this context, the review focuses on 3 key aspects: the study’s purpose, experimental design, and attributes of the investigational agent. This framework is designed to help researchers systematically assess appeal to participants at the time of enrollment, and retrospectively to evaluate the return on investment. The aim of this framework is to assist investigators in evaluating whether clinical trials with varying levels of return on investment are equitably enrolling different patient populations.

## Introduction

While several analyses suggest a survival advantage for trial participants,^1–3^ evidence supporting superior outcomes for cancer patients in clinical trials remains inconclusive.^2^^,^^4–6^ Aggregate assessment of trials obscures two truths: (1) individual participants derive diverse outcomes compared to that anticipated from standard care therapy^6^^,^^7^ and (2) individual trials provide diverse outcomes compared to standard therapy. Although qualitative studies have explored why patients participate in clinical trials, quantitative data on the value of participation in clinical trials are limited.^8^^,^^9^

**Figure 1.**
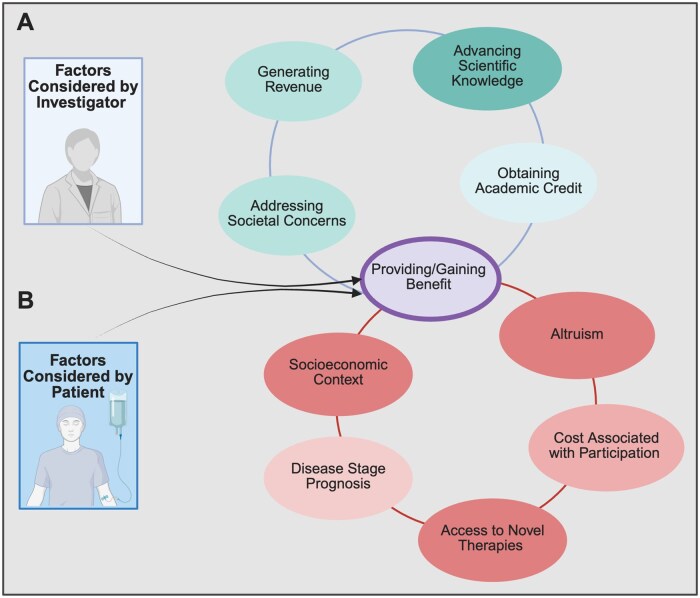
Factors considered by investigators and patients when offering or participating in clinical trials. (A) Depicts factors considered by investigators. Panel (B) shows considerations for patients. Both panels converge at the purple ellipse, symbolizing the shared interest of providing or gaining benefit for the participant.

Assessing the potential value for participants is particularly important at a time when the clinical research infrastructure increasingly focuses on equity.^10^ Data suggest that patients seeking cancer treatment mostly care about both quantity and quality of life.^11^ As such, offering trials that provide patients access to the newest available treatments is a primary motivation for investigators. Other reasons that investigators may consider for offering a clinical trial include advancing scientific knowledge, addressing societal concerns such as cost, generating clinical trial revenue, and obtaining academic credit^10^^,^^12–14^ (Figure 1A). Our group showed that studies for which investigators absorbed consent document translation costs were less likely to enroll patients with limited English proficiency.^15^ This suggests that the type of studies offered to potential trial participants may differ among distinct populations.

When deciding to participate in clinical trials, when one is available and offered, patients must weigh numerous factors. However, their decisions are often influenced more by the perceived potential benefits of participation rather than the actual benefits the trial may provide, which is generally unknown at the time of consent.^16^ While this could be similar for evaluating whether to pursue standard of care therapies, standard of care therapies have already been shown to be effective and safe, while investigational agents have not.

Factors that patients may consider in their decision-making when evaluating the risks and benefits of participating in a clinical trial include their disease characteristics, such as stage and prognosis, as well as their socioeconomic context^17^ (Figure 1B). Common incentives for participation may include access to novel interventions, potential therapeutic gain, obtaining expensive drugs at no cost, and receiving comprehensive medical care with frequent follow-ups and supportive services.^18–22^ However, these may come at the cost of additional responsibilities, such as frequent travel to trial sites, numerous laboratory tests, and potential unwarranted medical procedures, all of which can increase the financial and time demands placed on participants.^23^ Altruism and the desire to contribute to science may motivate some patients to participate in trials despite these burdens.^21^^,^^24^ Conversely, the primary reason patients decline trial participation is the sense of loss of control of their treatment choice.^17^ Furthermore, patients’ financial capacity to participate in a trial may also be highly influenced by extant economic conditions.^25^ Given the inherent opportunity costs that patients face when choosing between standard therapies and clinical trial participation, it is essential to establish a standardized framework to assess trials based on their return on investment, especially amidst efforts to improve enrollment to trials across different patient populations.^26^

In this review, we propose an initial framework for assessing the appeal and return on investment to clinical trial participants based on the characteristics of oncology drug development studies. The framework focuses on 3 main aspects: the study purpose, experimental design, and investigational agent. Prospectively, the framework assesses the potential appeal to participants by considering the study purpose and design, and some aspects of the investigational agent. Retrospectively, the framework assesses the return on investment, defined as the benefits derived from the investigational agent and having received enhanced medical care as a part of the clinical trial, even if participation required personal sacrifices such as time and travel, by incorporating attributes of the investigational agent.^27^ We hypothesize that patient demographics influence access to clinical trials, with more affluent individuals potentially enrolling in trials offering greater perceived benefit or return on investment. This may be due to their ability to absorb ancillary costs (eg, travel), greater English proficiency, or better access to information. However, this disparity has not been formally evaluated. Developing a standardized framework is a critical first step to assess whether trials with higher anticipated benefits systematically enroll patients from more privileged backgrounds.

Examples for each type of trial purpose, design and agent are described in the supplementary material section.

## Understanding patient motivators and fears about clinical trial participation

Research on patient participation in clinical trials reveals a complex interplay of factors influencing decision-making, often drawn from heterogeneous qualitative studies exploring patients’ experiences, perceptions, and motivations.^28^^,^^29^ A qualitative meta-analysis identified 6 key factors that may affect a patient’s willingness to participate in a study: perceptions of family, trust in physicians, hope for therapeutic benefit, altruism, availability of other treatment options, and cancer coping mechanism.^8^ While some patients may view trial participation as a financial necessity, others may recognize broader benefits, including societal contributions and personal health improvements.^30^ Studies also highlight that participants value the opportunity to advance science, improve patient care, and access high-quality medical treatment and expert care.^20^ However, the patient’s perception of personal benefit seems to be a key factor to participation.^17^^,^^31^

Studies show that when deciding to participate in trials, patients weigh different clinical trials characteristics in a positive or negative manner.^31^ For instance, patients often view randomization and dose-finding studies negatively, particularly when a new agent is tested as the first treatment option^28^^,^^31^ Similarly, the presence of a placebo group, lack of a control group, and blinding may deter patients from enrolling in trials.^31^^,^^32^ However, the concepts of randomization and blinding are complex and difficult for patients to comprehend.^33^ Patients with greater health literacy may better understand and weigh the risks and benefits of trial designs, such as randomization or the presence of a placebo, leading to more selective participation of these patients in trials that could have greater appeal or return on investment.

## Clinical trial features and perceived appeal or return on investment to participant

### Study purpose

Interventional study designs target specific research objectives. Dose-finding studies are designed to determine the highest tolerable dose with acceptable toxicity, often using dose-escalation methods.^34^ Because these trials often aim to establish the maximum tolerated dose rather than to establish efficacy, they may offer less direct patient benefit and inconsistent survival advantages.^35–37^ Conversely, proof-of-concept studies evaluate an agent’s efficacy and safety to determine its potential for evaluation in a larger trial in a specific population.^36^ These studies offer patients novel treatment approaches with pre-established doses that are hypothesized to provide benefits over standard of care therapies. The success of proof-of-concept studies is far from assured,^38^ as a review of 640 phase III trials for novel therapeutics found that 54% failed during late-stage development.^39^ Lastly, registration studies aim to establish a statistically significant difference in the efficacy of the investigated agent compared to the standard of care, facilitating regulatory approval.^40^ While these studies aim to investigate an active agent that has been proven safe in prior studies, from a patient’s perspective, concerns about participating in registration studies include the risk of being randomized, not knowing their treatment allocation, or being assigned to a placebo group.^41^

Other types of study designs include non-inferiority (NI) trials and biosimilar studies. NIs aim to show that a new treatment is not significantly worse than the standard of care by comparing it to a predefined efficacy margin.^42^^,^^43^ While not designed to prove superiority, the new therapy should ideally offer benefits such as cost-effectiveness, convenience, reduced invasiveness, or lower toxicity.^44^ Biosimilars studies, akin to NI trials, are important in expanding treatment options and reducing healthcare costs.^45^^,^^46^ Similarly, a challenge in biosimilar development is immunogenicity, which can provoke immune responses, increasing toxicity and reducing efficacy.^47^ Therefore, the benefits for participants in biosimilar trials may not always outweigh the risks.^48^ As such, since these studies are not designed to prove the superiority of a novel agent, they may not be perceived as appealing, and their return on investment is less certain.

### Experimental design

**Figure 2.**
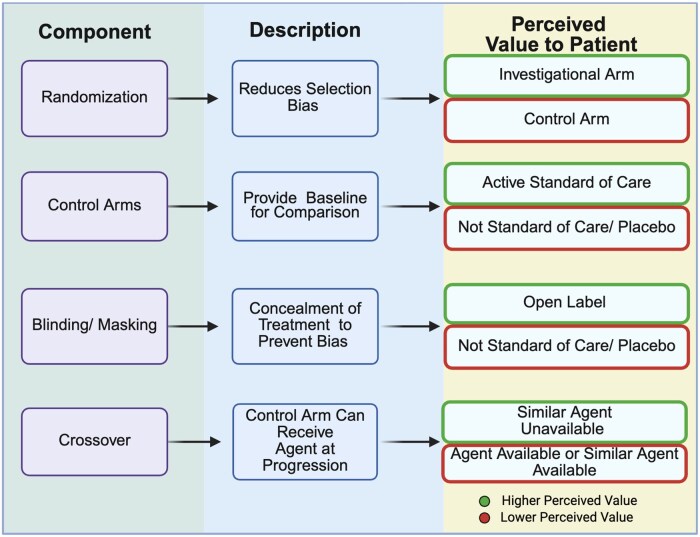
Key components of study design in assessing appeal or return on investment. The first column outlines the primary study design elements. The second column outlines the description of each study design component. The third column notes features of greater perceived value shown at the top and lesser perceived shown at the bottom.

Figure 2 shows an overview of different issues regarding the study design.

Certain aspects of the experimental design of a trial could make it more or less appealing or provide a higher return on investment. Randomization, which reduces the selection bias between study arms, introduces the possibility—often a 50% chance—that participants may not receive the novel agent being tested. From a scientific perspective, randomization is one of the methodological pillars of well-designed clinical trials, but is only ethical in a setting where equipoise as to which treatment is better can be reasonably determined to apply.^49^ Studies show that patients frequently view the prospect of being randomized to a control arm in a clinical trial as potentially harmful.^50^ Aversion to randomization has been reported as one of the primary reasons for trial participation refusal in various studies.^51^

As a crossover would eventually allow a patient on the control arm access to the investigational agent, this could be considered a positive attribute of a randomized clinical trial. When the investigational agent is already approved, crossover in a clinical trial would be the already established standard of care.^37^ A clinical trial that incorporates crossover upon progression could help ease the fear of randomization as participants would have confidence that they will receive the experimental agent during the course of their disease.^51^ Of note, although crossover can be a positive attribute from a patient’s perspective, frequently participants are not eligible to crossover based on worsening of laboratory values or clinical condition while the patient is receiving control arm therapy.^52^

While blinding safeguards against potential biases,^53^^,^^54^ the uncertainty associated with blinding may generate discomfort or dissatisfaction, particularly for individuals who value transparency in their medical care.^55^ Qualitative evidence has shown that blinding can lead to patient concerns about potential side effects or adverse reactions, as patients are not fully informed about the specific treatment they are receiving,^56^^,^^57^ resulting in heightened anxiety or apprehension, especially when patients are unable to anticipate or understand the potential outcomes of their treatment.^55^^,^^58^

Lastly, the control group on a clinical trial could alter the perception of appeal or return on investment from trial participation. For instance, control arms that do not mirror current standard treatments can potentially subject control group patients to suboptimal care, while casting doubt on the real advantages for patients in the experimental group.^59^^,^^60^ Although the Food and Drug Administration (FDA) generally requires studies to employ what is considered the standard of care at the initiation of the study, that standard can change over the course of the study, sometimes leading to a control arm that was demonstrated to be inferior to an approach approved after the study was initiated.^61^^,^^62^ Placebo, while uncommon in cancer clinical trials, can be administered if there is no clear standard of care at the time in which the study is being conducted.

### Investigated agent and subsequent approval

**Figure 3.**
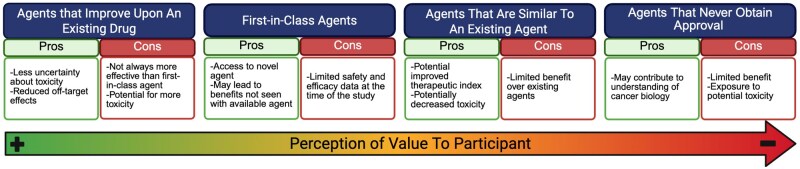
Pros and cons of different classes of investigational agents by retrospective analysis in clinical Trials. Arrow from left to right indicates classes of agents with less perceived value (left) to higher perceived value (right). For each class of agent, pros and cons of participation in a study based on retrospective assessment of the agent from a participant perspective is shown.

The return on investment to participants from receiving a particular investigational agent in a clinical trial is easier to assess retrospectively, as it is dependent on the agent’s efficacy, which is determined after the trial concludes. However, when therapies are already approved in a different tumor type or treatment setting, these could be considered as potentially more promising, and as such could be assessed in a prospective fashion (Figure 3).

First-in-class drugs are pharmaceutical agents with novel mechanisms of action that are thought to be innovative and can lead to unprecedented patient outcomes.^63^

One of the potential appeals of participating in trials is the ability to access innovative, first-in-class drugs. While these agents could be revolutionary and lead to changes in the standard of care, there is often minimal data regarding safety and efficacy at the time in which they are being studied.^64^ On the other hand, agents that improve upon previously available ones or that are already proven effective in other indications may have a similar mechanism of action and therapeutic indications as first-in-class agents, but are designed to enhance the efficacy and/or tolerability of existing drugs.^65^ As such, participating in studies evaluating such agents could lead to significant patient benefits with potentially less uncertainty about the potential efficacy and toxicity, and would allow to evaluate the appeal of the agent in a prospective fashion.^65^

Unlike first-in-class agents, “me-too” drugs are often structurally similar to first-in-class agents and developed to treat the same condition.^65^ Biosimilar agents fall into this category. Unlike biosimilar studies, some studies are designed to show the efficacy of a drug rather than similarity to an available drug, often the same comparator as the first-in-class agent. Perhaps unsurprisingly, enrollment from these studies generally disproportionately comes from places with limited practical availability of the existing agent, based on either regulatory or financial impediments.^66^

Lastly, numerous anti-cancer agents undergo extensive research but ultimately fail to secure FDA approval.^67^ For patients, participating in a study that ultimately fails to gain approval may not provide significant value. Some agents clearly offer benefit to a subgroup of patients but ultimately never end up being approved by the FDA. Notably, there is significant variability between countries regarding regulatory approval, and therefore, a lack of approval does not necessarily mean that patients may not experience benefit.

### A framework to objectively evaluate appeal and return on investment to study participants

While prior studies have been critical in elucidating patient perspectives and outcomes associated with clinical trial participation, they do not offer a systematic, broadly applicable method to evaluate the potential appeal or return on investment for trial participants.^49^^,^^50^^,^^68^ We propose a framework designed to retrospectively and prospectively benchmark characteristics of interventional clinical trials, specifically those evaluating pharmacological agents, that enhance the appeal or return on investment to participants. As trials entail a significant time investment for participants, this framework aims to systematically assess various components of clinical trials to determine which characteristics contribute most significantly to enhance the appeal or return on investment for participants.

**Figure 4.**
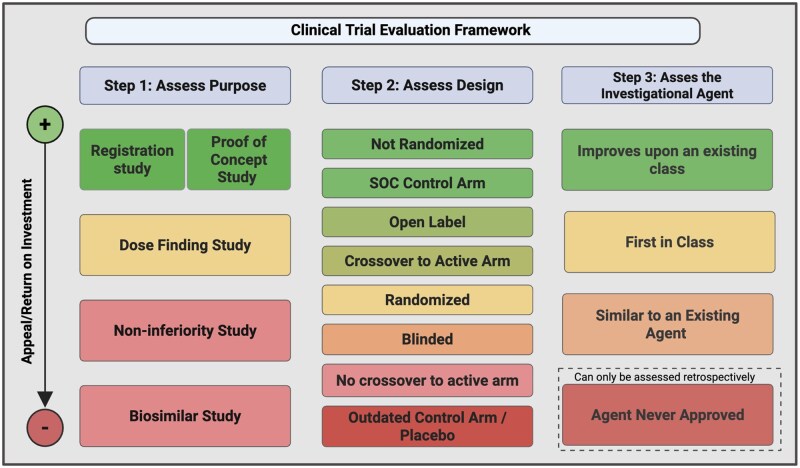
Clinical trial evaluation framework: assessing study purpose, design, and investigational agent. Abbreviation: SOC: standard of care. This framework assesses return on investment or appeal in 3 steps. The first step assesses trial purpose, ranging from registration or proof-of-concept studies (highest anticipated appeal, at the top) to biosimilar comparators (lowest anticipated appeal or return on investment, at the bottom). The second step evaluates study design, showing nonrandomized and studies with standard-of-care control arms (highest anticipated appeal or return on investment, at the top)) to outdated control or placebo arms (lowest anticipated appeal or retun on investment, at the bottom). The third step evaluates the investigational agent, agents that improve upon an existing class of agent(greatest perceived appeal or retun on investment at the top) to unapproved agents that can only be assessed retrospectively (lowest anticipated appeal or return on investement at the bottom).

**Figure 5.**
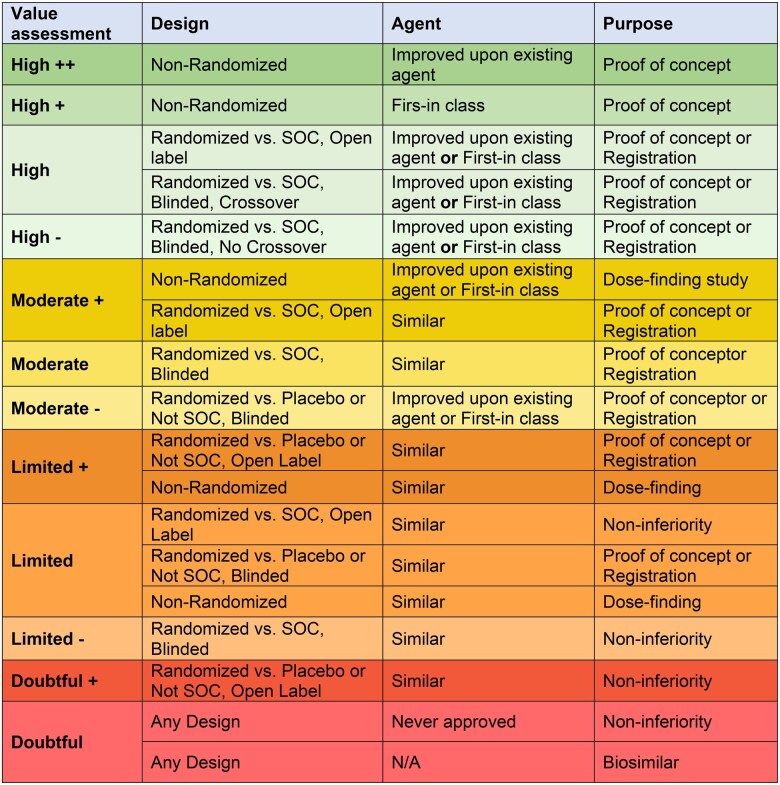
Clinical trial appeal or return on investment assessment. The clinical trial appeal/return on investment assessment takes into consideration the clinical trial framework to generate an appeal or return on investment scale to participants in trials. It divides studies from those that provide the highest appeal or return on investment at the topto studies with doubtful appeal or return on investment for participants at the bottom. Abbreviations: SOC; standard of care, NA; not applicable.

The proposed framework includes the 3 components described above, including the trial purpose, trial design, and investigational agent (Figure 4). Each of the components is assessed individually and then in the context of the other components. In such a way, investigators will be able to rank the study’s return on investment or appeal to participants (Figure 5). For instance, a non-randomized, proof-of-concept study evaluating an agent that is already approved in another setting or improves upon an existing type of agent may offer greater return on a patient’s time investment in terms of tangible outcomes like response and survival than an NI study assessing whether a new agent is not substantially worse than an existing standard.

This framework would serve as a starting point for assessing return on investment for patients who participated in studies and appeal to potential study participants. It is based on generalized assumptions on which study features provide greater benefit to participants, but it does not capture individual patient preferences, which can vary widely. A critical caveat is that most patients are not presented with multiple trial options from which to choose. In clinical practice, trial availability is often limited, and enrollment is influenced by factors beyond patient preference. Nevertheless, this framework may prove useful in identifying, among the available trials, those that offer comparatively greater potential benefit to participants.

### The importance of a framework to assess representativeness in clinical trial enrollment

There is no standardized framework to evaluate the appeal of clinical trials presented to potential trial participants or the return on investment to patients who participated in clinical trials. Understanding the potential appeal of clinical trials and the return on investment from having participated in them is crucial to ensure equitable access to groundbreaking studies that test agents that could become the new standard of care.

Equitable inclusion of historically underrepresented and vulnerable groups across all phases of clinical trials is crucial both for scientific validity and from a health equity perspective. Retrospective analysis of the types of studies in which historically underrepresented racial and ethnic groups are enrolled can help identify disparities and guide efforts to enhance their inclusion in trials that offer a higher return on investment to participants. The next step would be to apply the proposed framework to existing participant datasets to evaluate the demographic populations included in higher versus lower return on investment studies. Such an approach could, for instance, reveal that vulnerable populations are predominantly enrolled in studies with lower return on investment, such as NI or biosimilar trials.^69^^,^^70^ Furthermore, employing a prospective framework to evaluate the potential appeal to participants could help guarantee that diverse populations have access to trials with varying levels of appeal, fostering a more inclusive and balanced approach to clinical research^71^.

## Conclusion

Despite the existing body of research, a well-established and comprehensive framework defining characteristics of clinical trials that could have a greater or lower appeal or return on investment for participants is lacking. Such a framework is crucial for recognizing and benchmarking potential gaps in access to clinical trials deemed of appeal or return on investment from a patient’s perspective across different patient populations. The framework would represent an important tool for researchers and trial designers, enabling them to identify and overcome potential barriers to the inclusion of diverse patient populations across different studies.

## Supplementary Material

oyaf313_Supplementary_Data

## Data Availability

Does not apply.

## References

[1] Unger JM , Barlow WE, Martin DP, et al Comparison of survival outcomes among cancer patients treated in and out of clinical trials. J Natl Cancer Inst. 2014;106:dju002. 24627276 10.1093/jnci/dju002 PMC3982777

[2] Arrieta O , Carmona A, Ramírez-Tirado LA, et al Survival of patients with advanced non-small cell lung cancer enrolled in clinical trials. Oncology. 2016;91:185-193. 27449344 10.1159/000447404

[3] Merkhofer CM , Eaton KD, Martins RG, Ramsey SD, Goulart BHL. Impact of clinical trial participation on survival of patients with metastatic non-small cell lung cancer. Clin Lung Cancer. 2021;22:523-530. 34059474 10.1016/j.cllc.2021.04.003 PMC8531178

[4] Abu-Hejleh T , Chrischilles EA, Halfdanarson TR, et al The effect of receiving treatment within a clinical trial setting on survival and quality of care perception in advanced stage non–small cell lung cancer. Am J Clin Oncol. 2016;39:126-131. 24632817 10.1097/COC.0000000000000029 PMC4411190

[5] Iskander R , Moyer H, Vigneault K, Mahmud SM, Kimmelman J. Survival benefit associated with participation in clinical trials of anticancer drugs: a systematic review and meta-analysis. JAMA. 2024;331:2105–2113. 38767595 10.1001/jama.2024.6281 PMC11106715

[6] Fogel DB. Factors associated with clinical trials that fail and opportunities for improving the likelihood of success: A review. *Contemp Clin Trials Commun*. 2018;11:156-164. 10.1016/j.conctc.2018.08.00130112460 30112460 PMC6092479

[7] Gayet-Ageron A , Rudaz S, Perneger T. Study design factors influencing patients’ willingness to participate in clinical research: a randomised vignette-based study. BMC Med Res Methodol. 2020;20:93. 32336266 10.1186/s12874-020-00979-z PMC7183682

[8] Nielsen ZE , Berthelsen CB. Cancer patients’ perceptions of factors influencing their decisions on participation in clinical drug trials: a qualitative meta-synthesis. J Clin Nurs. 2019;28:2443-2461. 30673153 10.1111/jocn.14785

[9] Moorcraft SY , Marriott C, Peckitt C, et al Patients’ willingness to participate in clinical trials and their views on aspects of cancer research: results of a prospective patient survey. Trials. 2016;17:17. 26745891 10.1186/s13063-015-1105-3 PMC4706669

[10] Acuña-Villaorduña A , Baranda JC, Boehmer J, Fashoyin-Aje L, Gore SD. Equitable access to clinical trials: how do we achieve it? Am Soc Clin Oncol Educ Book. 2023;43:e389838. 37146264 10.1200/EDBK_389838

[11] Samuel JN , Booth CM, Eisenhauer E, Brundage M, Berry SR, Gyawali B. Association of quality-of-life outcomes in cancer drug trials with survival outcomes and drug class. JAMA Oncol. 2022;8:879-886. 35482347 10.1001/jamaoncol.2022.0864 PMC9052107

[12] Messner DA , Moloney R, Warriner AH, Wright NC, Foster PJ, Saag KG. Understanding practice-based research participation: the differing motivations of engaged vs. non-engaged clinicians in pragmatic clinical trials. Contemp Clin Trials Commun. 2016;4:136-140. 29736476 10.1016/j.conctc.2016.08.003 PMC5935887

[13] Al-Shami KM , Ahmed WS, Alzoubi KH. Motivators and barriers towards clinical research participation: a population-based survey from an Arab MENA country. Kerasidou A, editor. PLoS One. 2022;17:e0270300. 35749422 10.1371/journal.pone.0270300 PMC9231817

[14] Mahmud A , Zalay O, Springer A, Arts K, Eisenhauer E. Barriers to participation in clinical trials: a physician survey. Curr Oncol. 2018;25:119-125. 29719427 10.3747/co.25.3857 PMC5927782

[15] Velez MA , Glenn BA, Garcia-Jimenez M, et al Consent document translation expense hinders inclusive clinical trial enrolment. Nature. 2023;620:855-862. 37532930 10.1038/s41586-023-06382-0 PMC11046417

[16] Lim Y , Lim JM, Jeong WJ, et al Korean cancer patients’ awareness of clinical trials, perceptions on the benefit and willingness to participate. Cancer Res Treat. 2017;49:1033-1043. 28392549 10.4143/crt.2016.413 PMC5654169

[17] Unger JM , Hershman DL, Albain KS, et al Patient income level and cancer clinical trial participation. J Clin Oncol. 2013;31:536-542. 23295802 10.1200/JCO.2012.45.4553 PMC3565180

[18] González-González JG , González-Saldivar G, Rodríguez-Gutiérrez R. Participants perception of pharmaceutical clinical research: a cross-sectional controlled study. Patient Prefer Adherence. 2016;727-734. 10.2147/PPA.S96021 PMC4857804 27199549

[19] Ellis PM , Butow PN, Tattersall MH, Dunn SM, Houssami N. Randomized clinical trials in oncology: understanding and attitudes predict willingness to participate. *J Clin Oncol*. 2001;19:3554-3561. 10.1200/JCO.2001.19.15.355411481363 11481363

[20] Willison DJ , Richards DP, Orth A, Harris H, Marlin S. Survey of awareness and perceptions of Canadians on the benefits and risks of clinical trials. Ther Innov Regul Sci. 2019;53:669-677. 30373453 10.1177/2168479018805433 PMC6710611

[21] Jenkins V , Fallowfield L. Reasons for accepting or declining to participate in randomized clinical trials for cancer therapy. Br J Cancer. 2000;82:1783-1788. 10839291 10.1054/bjoc.2000.1142 PMC2363224

[22] Coyne CA , Xu R, Raich P, et al Randomized, controlled trial of an easy-to-read informed consent statement for clinical trial participation: a study of the Eastern Cooperative Oncology Group. J Clin Oncol. 2003;21:836-842. 12610182 10.1200/JCO.2003.07.022

[23] McKinney M , Bell R, Samborski C, et al Clinical trial participation: a pilot study of patient-identified barriers. Clin J Oncol Nurs. 2021;25:647-654. 34800100 10.1188/21.CJON.647-654 PMC10150445

[24] Unger JM , Cook E, Tai E, Bleyer A. The role of clinical trial participation in cancer research: barriers, evidence, and strategies. Am Soc Clin Oncol Educ Book. 2016;35:185-198. 27249699 10.14694/EDBK_156686 PMC5495113

[25] Unger JM , Xiao H, Vaidya R, LeBlanc M, Hershman DL. Medicaid expansion of the patient protection and affordable care act and participation of patients with Medicaid in cancer clinical trials. JAMA Oncol. 2023;9:1371-1379. 37590003 10.1001/jamaoncol.2023.2800 PMC10436183

[26] Ford JG , Howerton MW, Lai GY, et al Barriers to recruiting underrepresented populations to cancer clinical trials: a systematic review. Cancer. 2008;112:228-242. 18008363 10.1002/cncr.23157

[27] Nhlbi N. *Clinical Trials Benefits, Risks, and Safety Measures*. Accessed 10/05/2025 https://www.nhlbi.nih.gov/research/clinical-trials/safety-benefits-risks#:∼:text=You%20gain%20access%20to%20new, doctors%20and%20other%20healthcare%20professionals.

[28] Madsen SM , Mirza MR, Holm S, Hilsted KL, Kampmann K, Riis P. Attitudes towards clinical research amongst participants and nonparticipants. *J Intern Med*. 2002;251:156-168. 10.1046/j.1365-2796.2002.00949.x11905591 11905591

[29] Kost RG , Lee LN, Yessis JL, et al Research participant-centered outcomes at NIH-supported clinical research centers: Research Participant Experience Outcomes. Clin Transl Sci. 2014;7:430-440. 24842076 10.1111/cts.12167 PMC4237675

[30] Fisher JA , McManus L, Wood MM, et al Healthy volunteers’ perceptions of the benefits of their participation in phase I clinical trials. J Empir Res Hum Res Ethics. 2018;13:494-510. 30296882 10.1177/1556264618804962 PMC6235676

[31] Dias AL , Chao JH, Lee D, Wu Y, Kloecker GH. Patient perceptions concerning clinical trials in oncology patients. Contemp Clin Trials Commun. 2016;4:179-185. 29736480 10.1016/j.conctc.2016.09.005 PMC5935901

[32] Dennstädt F , Putora PM, Iseli T, Treffers T, Panje C, Fischer GF. Patient autonomy and shared decision‐making in the context of clinical trial participation. Eur J Clin Invest. 2024;54:e14291. 39086071 10.1111/eci.14291

[33] Mills EJ , Seely D, Rachlis B, et al Barriers to participation in clinical trials of cancer: a meta-analysis and systematic review of patient-reported factors. Lancet Oncol. 2006;7:141-148. 16455478 10.1016/S1470-2045(06)70576-9

[34] Scmidt R. Dose-finding studies in clinical drug development. Eur J Clin Pharmacol. 1988;34:15-19. 3360047 10.1007/BF01061410

[35] Peppercorn JM , Weeks JC, Cook EF, Joffe S. Comparison of outcomes in cancer patients treated within and outside clinical trials: conceptual framework and structured review. Lancet. 2004;363:263-270. 14751698 10.1016/S0140-6736(03)15383-4

[36] Bittlinger M , Bicer S, Peppercorn J, Kimmelman J. Ethical considerations for phase I trials in oncology. J Clin Oncol. 2022;40:3474-3488. 35275736 10.1200/JCO.21.02125

[37] Gyawali B , Bowman M, Sharpe I, Jalink M, Srivastava S, Wijeratne DT. A systematic review of eHealth technologies for breast cancer supportive care. Cancer Treat Rev. 2023;14:102519. 10.1016/j.ctrv.2023.102519 36736125

[38] Sun D , Gao W, Hu H, Zhou S. Why 90% of clinical drug development fails and how to improve it? Acta Pharm Sin B. 2022;12:3049-3062. 35865092 10.1016/j.apsb.2022.02.002 PMC9293739

[39] Hwang TJ , Carpenter D, Lauffenburger JC, Wang B, Franklin JM, Kesselheim AS. Failure of investigational drugs in late-stage clinical development and publication of trial results. JAMA Intern Med. 2016;176:1826-1833. 27723879 10.1001/jamainternmed.2016.6008

[40] Li QH , Deng Q, Ting N. Proof of concept: drug selection? or dose selection? thoughts on multiplicity issues. Ther Innov Regul Sci. 2021;55:1001-1005. 34028669 10.1007/s43441-021-00299-4 PMC8142878

[41] Heneghan C , Goldacre B, Mahtani KR. Why clinical trial outcomes fail to translate into benefits for patients. Trials. 2017;18:122. 28288676 10.1186/s13063-017-1870-2 PMC5348914

[42] Mauri L , D’Agostino RB. Challenges in the design and interpretation of noninferiority trials. N Engl J Med. 2017;377:1357-1367. 28976859 10.1056/NEJMra1510063

[43] Haslam A , Gill J, Prasad V. An empirical analysis of noninferiority studies in oncology: are they good enough? J Natl Compr Canc Netw. 2020;18:161-167. 32023529 10.6004/jnccn.2019.7349

[44] Powers JH , Cooper CK, Lin D, Ross DB. Sample size and the ethics of non-inferiority trials. Lancet. 2005;366:24-25. 10.1016/S0140-6736(05)66817-1 15993221

[45] Joshi D , Khursheed R, Gupta S, et al Biosimilars in oncology: latest trends and regulatory status. Pharmaceutics. 2022;14:2721. 36559215 10.3390/pharmaceutics14122721 PMC9784530

[46] Nahleh Z , Lyman GH, Schilsky RL, et al Use of biosimilar medications in oncology. JCO Oncol Pract. 2022;18:177-186. 35041524 10.1200/OP.21.00771

[47] Yoo C , Im H-S, Kim K-P, et al Real-world efficacy and safety of liposomal irinotecan plus fluorouracil/leucovorin in patients with metastatic pancreatic adenocarcinoma: a study by the Korean Cancer Study Group. Ther Adv Med Oncol. 2019;11:1758835919871126. 31489036 10.1177/1758835919871126 PMC6710683

[48] Ohn JA , Atteberry PJ, Trusheim MR, Bach PB. Ethical and human subject burdens of trials conducted to evaluate biosimilars. Health Policy. 2021. 10.1101/2021.03.05.21252938

[49] Ulrich CM , Knafl KA, Ratcliffe SJ, et al Developing a model of the benefits and burdens of research participation in cancer clinical trials. AJOB Prim Res. 2012;3:10-23. 24748992 10.1080/21507716.2011.653472 PMC3989990

[50] Naidoo N , Nguyen VT, Ravaud P, et al The research burden of randomized controlled trial participation: a systematic thematic synthesis of qualitative evidence. BMC Med. 2020;18:6. 31955710 10.1186/s12916-019-1476-5 PMC6970283

[51] Llewellyn-Thomas HA , McGreal MJ, Thiel EC, Fine S, Erlichman C. Patients’ willingness to enter clinical trials: measuring the association with perceived benefit and preference for decision participation. Soc Sci Med. 1991;32:35-42. 2008619 10.1016/0277-9536(91)90124-u

[52] Haslam A , Prasad V. When is crossover desirable in cancer drug trials and when is it problematic? Ann Oncol. 2018;29:1079-1081. 29648572 10.1093/annonc/mdy116 PMC5961160

[53] Strite SA , Stuart ME. Importance of blinding in randomized trials. JAMA. 2010;304:2127-2128; author reply 2128. 10.1001/jama.2010.1621 21081725

[54] Betensky RA. Don’t be blinded by the blinding. NEJM Evid. 2022;1. 10.1056/EVIDe2100063 38319191

[55] Wan M , Orlu-Gul M, Legay H, Tuleu C. Blinding in pharmacological trials: the devil is in the details. Arch Dis Child. 2013;98:656-659. 23898156 10.1136/archdischild-2013-304037 PMC3833301

[56] Crisp A. Blinding in pharmaceutical clinical trials: an overview of points to consider. Contemp Clin Trials. 2015;43:155-163. 26044462 10.1016/j.cct.2015.06.002

[57] Anand R , Norrie J, Bradley JM, McAuley DF, Clarke M. Fool’s gold? Why blinded trials are not always best. BMJ. 2020;368:l6228. 31964628 10.1136/bmj.l6228

[58] Monaghan TF , Agudelo CW, Rahman SN, et al Blinding in clinical trials: seeing the big picture. Medicina (Kaunas). 2021;57:647. 34202486 10.3390/medicina57070647 PMC8308085

[59] Rossi A , Aimar G, Audisio M, et al Analysis of the adequacy of control arms in oncology randomised clinical trials published between 2017 and 2021: a meta-research study. Eur J Cancer. 2023;189:112920. 37277262 10.1016/j.ejca.2023.05.008

[60] Hilal T , Sonbol MB, Prasad V. Analysis of control arm quality in randomized clinical trials leading to anticancer drug approval by the US Food and Drug Administration. JAMA Oncol. 2019;5:887-892. 31046071 10.1001/jamaoncol.2019.0167 PMC6499129

[61] Del Paggio JC , Berry JS, Hopman WM, et al Evolution of the randomized clinical trial in the era of precision oncology. JAMA Oncol. 2021;7:728-734. 33764385 10.1001/jamaoncol.2021.0379 PMC7995135

[62] Benjamin DJ , Xu A, Lythgoe MP, Prasad V. Cancer drug approvals that displaced existing standard-of-care therapies, 2016-2021. JAMA Netw Open. 2022;55:e222265. 35289858 10.1001/jamanetworkopen.2022.2265 PMC8924721

[63] Center for Drug Evaluation and Research. U.S. Food and Drug Administration. Novel Drug Approvals for 2023. n.d. Accessed May 10, 2025. https://www.fda.gov/drugs/novel-drug-approvals-fda/novel-drug-approvals-2023

[64] Lexchin J. Health Canada’s use of expedited review pathways and therapeutic innovation, 1995-2016: cross-sectional analysis. BMJ Open. 2018;8:e023605. 10.1136/bmjopen-2018-023605 PMC6119438 30166310

[65] Aronson JK , Green AR. Me‐too pharmaceutical products: history, definitions, examples, and relevance to drug shortages and essential medicines lists. Br J Clin Pharmacol. 2020;86:2114-2122. 32358800 10.1111/bcp.14327 PMC7576625

[66] Ali S , Egunsola O, Babar ZUD, Hasan SS. Clinical trials in Asia: a World Health Organization database study. Perspect Clin Res. 2019;10:121-124. 31404203 10.4103/picr.PICR_109_18 PMC6647899

[67] FDA U.S. Food and Drug Administration. Withdrawn | Cancer Accelerated Approvals [Internet]. FDA; 2024. Accessed May 10, 2025. https://www.fda.gov/drugs/resources-information-approved-drugs/withdrawn-cancer-accelerated-approvals

[68] Ulrich CM , Ratcliffe SJ, Zhou Q, et al Association of perceived benefit or burden of research participation with participants’ withdrawal from cancer clinical trials. JAMA Netw Open. 2022;5:e2244412. 36449287 10.1001/jamanetworkopen.2022.44412 PMC9713607

[69] Allison K , Patel D, Kaur R. Assessing multiple factors affecting minority participation in clinical trials: development of the clinical trials participation barriers survey. Cureus. 2022;14::e24424. 35637812 10.7759/cureus.24424 PMC9127181

[70] Hamel LM , Penner LA, Albrecht TL, Heath E, Gwede CK, Eggly S. Barriers to clinical trial enrollment in racial and ethnic minority patients with cancer. Cancer Control. 2016;23:327-337. 27842322 10.1177/107327481602300404 PMC5131730

[71] Umscheid CA , Margolis DJ, Grossman CE. Key concepts of clinical trials: a narrative review. Postgrad Med. 2011;123:194-204. 21904102 10.3810/pgm.2011.09.2475 PMC3272827

