# Supplementary material for: Characteristics of clinical trials associated with appeal and return on investment to participants: a review and framework
Source: Oncologist. 2025 Sep 27;30(10):oyaf313. doi: 10.1093/oncolo/oyaf313 (PMC12578512; doi:10.1093/oncolo/oyaf313)
Supplement: oyaf313_Supplementary_Data [file oyaf313_supplementary_data.docx]

**Supplementary Information**

**Dose-Finding Studies:**

Examples: Some agents can have both efficacy and minimal toxicity at low doses. In patients with gastrointestinal stromal tumors (GIST), imatinib at both 400 mg and 600 mg daily showed comparable response rates, median progression-free survival (PFS), and median overall survival (OS) with limited toxicity(1). Conversely, busulfan, a potent alkylating agent commonly used in conditioning regimens for hematopoietic stem cell transplantation, has a narrow therapeutic index(1). Studies indicate that subtherapeutic exposure increases the risk of relapse and compromises OS, while supratherapeutic concentrations elevate toxicity and diminish OS(2).

**Proof-of-concept studies**

Examples: Dostarlimab, an immune checkpoint inhibitor, was evaluated in a phase II study of patients with stage II or III rectal carcinoma and mismatch repair deficiency, resulting in complete responses and minimal toxicity(3). In contrast, rovalpituzumab tesirine, an antibody drug conjugate for second-line small cell lung cancer with high DLL3 expression, showed initial responses but had its development halted in a later study due to lower survival compared to the control arm(4). The toxicity of the agent at the selected dose was substantial and hypothesized to drive the unfavorable outcome. In retrospect, participants in this proof-of-concept trial would have experienced greater efficacy and lower toxicity with the standard of care therapy.

**Registration Studies**

Examples: Patient perspectives in a registration study are illustrated by a randomized open-label clinical trial of Lutetium-177, a radioligand therapy, for metastatic castration-resistant prostate cancer(5). Participants were randomized to receive Lutetium-177 plus standard of care versus standard of care alone. Attrition rates were higher in the control arm(6), suggesting that patients randomized to the control arm left the study out of dissatisfaction with not receiving the novel agent.

**Non-inferiority Studies**

Examples: Non-inferiority trials in cancer research that showed no long-term benefit, despite initially non-inferior results, are the ones comparing bevacizumab with standard chemotherapy for metastatic breast cancer(7,8) . Follow-up studies revealed no significant improvement in overall survival for patients treated with bevacizumab compared to standard chemotherapy alone(9). Results revealed that benefits in PFS did not translate into a meaningful increase in quality of life or long-term outcomes(10).

**Biosimilar Studies**

Examples: A post market study evaluating filgrastim analogues showed that biosimilar agents had higher rates of adverse effects such as pyrexia, myalgia and neutropenia, than those reported with the use of the original agent(11). It should be acknowledged that for this class of drugs, data showing inferiority of biosimilar agents needs to recognize that the makers of the generally more expensive comparator agent have a substantial incentive to cast doubt of the similarity of the biosimilar agent, complicating efforts to generate objective data.

**Randomization**

Examples: A study of patients with BRAF V600E mutation-positive melanoma randomized participants to receive either dabrafenib, a targeted agent, or the chemotherapeutic dacarbazine(12). Dacarbazine is difficult to tolerate, with a response rate of about 20% and a median survival of 6-9 months(13). At the time of the study, enough data was available to make randomization to dacarbazine unattractive to potential participants.

**Selection of Control Condition**

Example: The POLO trial evaluated maintenance olaparib, a PARP inhibitor, in patients with metastatic pancreatic cancer and germline BRCA mutations(14). Patients in the control group received placebo when chemotherapy was considered the standard of care within the community(15).

**Blinding/Masking**

Examples:

In the double-blind adjuvant trial comparing tamoxifen to placebo, patients randomized to the placebo group may have faced significant dissatisfaction, particularly in light of the trial's findings(16). The study involved 181 elderly women with node-positive breast cancer and demonstrated that tamoxifen significantly extended the median time to treatment failure—7.4 years for tamoxifen compared to 4.4 years for placebo—while also reducing recurrence and mortality rates(16). Participants assigned to placebo may have been frustrated upon learning they did not receive a treatment that could have greatly improved their prognosis and survival outcomes.

**Crossover Studies**

Examples: The epidermal growth factor receptor (EGFR) tyrosine kinase inhibitor (TKI) gefinitib was evaluated against chemotherapy in a randomized controlled trial that included patients with lung cancer and *EGFR* sensitizing mutations(17). Despite a significant improvement in progression free survival (PFS), there was not a statisically siginificant difference in OS between the two groups, possibly secondary to crossover, as around to 95% of patients in the chemotherapy arm crossed over to receive gefitinib upon disease progression.

**First-in-class agents**

Examples: In the initial phase I study of imatinib, 97% of patients achieved a complete response(18). These patients were the first to benefit from a revolutionary treatment for patients with chronic myeloid leukemia. However, they took the risk of trying this new therapy, uncertain if the potential benefits would be significant or relevant to their condition(18,19). Similarly, patients with NSCLC and melanoma who participated in the KEYNOTE-001 study and received pembrolizumab stood to have significant and durable benefit from therapy with 5-year OS rates over 30% in patients with melanoma and close to 15% in patients with NSCLC(20,21).

**Agents that improve upon an existing class of drug**

Examples: The ALEX trial compared alectinib, a newer generation ALK TKI, to crizotinib in patients with *ALK*-rearranged metastatic NSCLC(22). Results from thus study showed that PFS was superior with alectinib, and incidence of grade 3 or 4 adverse events were lower. In addition, the effectiveness against brain metastases, a substantial clinical concern among these patients, was much greater with alectinib.

**Agents that are similar to an existing agent:**

Examples: Although cemiplimab was first in its class agent for the treatment of cutaneous squamous cell carcinoma, it was tested against chemotherapy in patients with metastatic NSCLC, at a time when pembrolizumab, a similar agent, was already approved in this patient population(23). PFS and OS were superior in the cemiplimab arm, but these improvements were comparable to those seen with pembrolizumab with similar efficacy and safety(24–26).

**Agents that are never approved:**

Examples: Rociletinib, a third-generation EGFR TKI with potent activity against *EGFR*^T790M^ initially demonstrated efficacy in a phase 1-2 study evaluated the efficacy of in patients with previously treated metastatic NSCLC with an ORR of 59% (95%CI 45-73)(27). However, the rates of hyperglycemia and QTc prolongation were high. Because of its lower efficacy and higher toxicity rates as compared to the contemporaneously approved osimertinib, its development was halted, and the agent was never approved.

**Dose-Finding Studies:**

| Drug | Class | Indication | Key Findings |
| --- | --- | --- | --- |
| Imatinib | Tyrosine Kinase Inhibitors | GIST | Comparable response rates and median PFS/OS at 400 mg and 600 mg with limited toxicity (19) |
| Busulfan | Alkylating Agent | Hematopoietic Stem Cell Transplantation | Narrow therapeutic index; subtherapeutic doses increase relapse risk, while supratherapeutic doses elevate toxicity (1,2) |

**Proof-of-concept studies**

| Drug | Class | Indication | Key Findings |
| --- | --- | --- | --- |
| Dostarlimab | Immune Checkpoint Inhibitor | Rectal Carcinoma | Evaluated in a phase II study with complete responses and minimal toxicity in stage II or III with mismatch repair deficiency(3). |
| Rovalpituzumab Tesirine | Antibody-Drug Conjugate | Small  Cell  Lung Cancer | Showed initial responses, but development halted due to lower survival compared to the control arm and substantial toxicity(4). |

**Registration Studies**

| Drug | Class | Indication | Key Findings |
| --- | --- | --- | --- |
| Lutetium-177 | Radioligand | Prostate Cancer | Evaluated in a randomized open-label trial; higher attrition rates in the control arm indicate dissatisfaction among patients not receiving the novel agent(5,6). |

**Non-inferiority Studies**

| Drug | Class | Indication | Key Findings |
| --- | --- | --- | --- |
| Bevacizumab vs Conventional Chemotherapy | Monoclonal Antibody against VEGF | Breast Cancer | Non-inferiority trials showed no long-term benefit(9,10); no significant improvement in overall survival or quality of life compared to standard chemotherapy. |

**Biosimilar Studies**

| Drug | Class | Indication | Key Findings |
| --- | --- | --- | --- |
| Filgrastim | Colony-Stimulating Factor | Febrile Neutropenia | Post-market study indicated higher rates of adverse effects with biosimilars compared to the original agent; concerns exist regarding bias from manufacturers of the more expensive comparators(11). |

**Randomization**

| Drug | Class | Indication | Key Findings |
| --- | --- | --- | --- |
| Dabrafenib | BRAF Inhibitors | Melanoma | Study showed participants randomized to dabrafenib versus dacarbazine; dacarbazine had a low response rate and was poorly tolerated, making it an unattractive option for potential participants(12,13). |

**Selection of Control Condition**

| Drug | Class | Indication | Key Findings |
| --- | --- | --- | --- |
| Olaparib | PARP Inhibitor | Metastatic Pancreatic Cancer | The POLO trial evaluated olaparib as maintenance therapy for patients with germline BRCA mutations; control group received placebo when standard of care was chemotherapy(14,15). |

**Blinding/Masking**

| Drug | Class | Indication | Key Findings |
| --- | --- | --- | --- |
| Tamoxifen | Selective Estrogen Receptor Modulators | Breast Cancer | In a double-blind trial, tamoxifen significantly extended median time to treatment failure (7.4 years vs. 4.4 years for placebo) and reduced recurrence and mortality, potentially leading to dissatisfaction among placebo participants(16). |

**Crossover Studies**

| Drug | Class | Indication | Key Findings |
| --- | --- | --- | --- |
| Gefitinib | Tyrosine Kinase Inhibitors | Lung Cancer | In a trial, gefitinib showed improved progression-free survival (PFS) compared to chemotherapy, but no significant difference in overall survival (OS) due to high crossover rates (95%) to gefitinib from the chemotherapy arm (17). |

**First-in-class agents**

| Drug | Class | Indication | Key Findings |
| --- | --- | --- | --- |
| Imatinib | Tyrosine Kinase Inhibitors | Chronic Myeloid Leukemia | In a phase I study, 97% of patients achieved a complete response, marking a revolutionary treatment, though they faced uncertainty about the significance of potential benefits(18,19). |
| Pembrolizumab | Immune Checkpoint Inhibitors | Non-Squamous Cell Lung Cancer and Melanoma | Patients in the KEYNOTE-001 study receiving pembrolizumab had significant benefits, with 5-year OS rates >30% in melanoma and nearly 15% in NSCLC(20,21). |

**Agents that improve upon an existing class of drug**

| Drug | Class | Indication | Key Findings |
| --- | --- | --- | --- |
| Alectinib | Tyrosine Kinase Inhibitors | Non-Squamous Cell Lung Cancer (ALK Negative) | The ALEX trial demonstrated that alectinib had superior progression-free survival (PFS) and lower rates of grade 3 or 4 adverse events compared to crizotinib, with enhanced effectiveness against brain metastases(22). |

**Agents that are similar to an existing agent:**

| Drug | Class | Indication | Key Findings |
| --- | --- | --- | --- |
| Cemiplimab | Immune Checkpoint Inhibitors | Cutaneous Squamous Cell Carcinoma | Cemiplimab was evaluated against chemotherapy for metastatic NSCLC and showed superior PFS and OS compared to chemotherapy(24), though improvements were comparable to pembrolizumab with similar efficacy and safety(25). |

**Agents that are never approved:**

| Drug | Class | Indication | Key Findings |
| --- | --- | --- | --- |
| Rociletinib | Tyrosine Kinase Inhibitors | Non-Squamous Cell  Lung Cancer | Rociletinib showed an objective response rate (ORR) of 59% in a phase 1-2 study for metastatic NSCLC, but high rates of hyperglycemia and QTc prolongation led to its development being halted due to lower efficacy and higher toxicity compared to Osimertinib(27). |

Supplementary Materials References

1. Krivoy N, Hoffer E, Lurie Y, Bentur Y, Rowe J. Busulfan Use in Hematopoietic Stem Cell Transplantation: Pharmacology, Dose Adjustment, Safety and Efficacy in Adults and Children. Curr Drug Saf. 2008 Jan 1;3(1):60–6.

2. Hoffer E, Akria L, Tabak A, Scherb I, Rowe JM, Krivoy N. A Simple Approximation for Busulfan Dose Adjustment in Adult Patients Undergoing Bone Marrow Transplantation: Ther Drug Monit. 2004 Jun;26(3):331–5.

3. Cercek A, Lumish M, Sinopoli J, Weiss J, Shia J, Lamendola-Essel M, et al. PD-1 Blockade in Mismatch Repair–Deficient, Locally Advanced Rectal Cancer. N Engl J Med. 2022 Jun 23;386(25):2363–76.

4. Blackhall F, Jao K, Greillier L, Cho BC, Penkov K, Reguart N, et al. Efficacy and Safety of Rovalpituzumab Tesirine Compared With Topotecan as Second-Line Therapy in DLL3-High SCLC: Results From the Phase 3 TAHOE Study. J Thorac Oncol. 2021 Sep;16(9):1547–58.

5. Olivier T, Powell K, Prasad V. Lutetium-177-PSMA-617 in Metastatic Castration-resistant Prostate Cancer: Limitations of the VISION Trial. Eur Urol. 2023 Jul;84(1):4–6.

6. Sartor O, De Bono J, Chi KN, Fizazi K, Herrmann K, Rahbar K, et al. Lutetium-177–PSMA-617 for Metastatic Castration-Resistant Prostate Cancer. N Engl J Med. 2021 Sep 16;385(12):1091–103.

7. Cameron D. Bevacizumab in the first-line treatment of metastatic breast cancer. Eur J Cancer Suppl. 2008 Mar;6(6):21–8.

8. Montero AJ, Escobar M, Lopes G, Glück S, Vogel C. Bevacizumab in the Treatment of Metastatic Breast Cancer: Friend or Foe? Curr Oncol Rep. 2012 Feb;14(1):1–11.

9. Brufsky A. Is there room for bevacizumab in metastatic breast cancer? Lancet Oncol. 2016 Sep;17(9):1175–6.

10. Mezzanotte-Sharpe J, ONeill A, Mayer IA, Arteaga CL, Yang XJ, Wagner LI, et al. A randomized phase III double-blind placebo-controlled trial of first line chemotherapy and trastuzumab with or without bevacizumab for patients with HER2/neu-positive metastatic breast cancer: a trial of the ECOG-ACRIN Cancer Research Group (E1105) [Internet]. 2024 [cited 2024 Sep 23]. Available from: https://www.researchsquare.com/article/rs-4295044/v1

11. Cornes PG, Muenzberg M. Commentary - A comprehensive safety understanding of granulocyte-colony stimulating factor biosimilars and Intended Copy Biologics in treating chemotherapy associated febrile neutropenia. Toxicol Appl Pharmacol. 2020 Nov;406:115202.

12. Hauschild A, Grob JJ, Demidov LV, Jouary T, Gutzmer R, Millward M, et al. Dabrafenib in BRAF-mutated metastatic melanoma: a multicentre, open-label, phase 3 randomised controlled trial. The Lancet. 2012 Jul;380(9839):358–65.

13. Serrone L, Zeuli M, Sega FM, Cognetti F. Dacarbazine-based chemotherapy for metastatic melanoma: thirty-year experience overview. J Exp Clin Cancer Res CR. 2000 Mar;19(1):21–34.

14. Golan T, Hammel P, Reni M, Van Cutsem E, Macarulla T, Hall MJ, et al. Maintenance Olaparib for Germline *BRCA* -Mutated Metastatic Pancreatic Cancer. N Engl J Med. 2019 Jul 25;381(4):317–27.

15. Nishikawa G, Booth C, Prasad V. Olaparib for BRCA mutant pancreas cancer: Should the POLO trial change clinical practice? Cancer. 2020 Sep 15;126(18):4087–8.

16. Cummings FJ, Gray R, Tormey DC, Davis TE, Volk H, Harris J, et al. Adjuvant tamoxifen versus placebo in elderly women with node-positive breast cancer: long-term follow-up and causes of death. J Clin Oncol. 1993 Jan;11(1):29–35.

17. Maemondo M, Inoue A, Kobayashi K, Sugawara S, Oizumi S, Isobe H, et al. Gefitinib or Chemotherapy for Non–Small-Cell Lung Cancer with Mutated EGFR. N Engl J Med. 2010 Jun 24;362(25):2380–8.

18. Druker BJ, Talpaz M, Resta DJ, Peng B, Buchdunger E, Ford JM, et al. Efficacy and Safety of a Specific Inhibitor of the BCR-ABL Tyrosine Kinase in Chronic Myeloid Leukemia. N Engl J Med. 2001 Apr 5;344(14):1031–7.

19. Druker BJ, Guilhot F, O’Brien SG, Gathmann I, Kantarjian H, Gattermann N, et al. Five-Year Follow-up of Patients Receiving Imatinib for Chronic Myeloid Leukemia. N Engl J Med. 2006 Dec 7;355(23):2408–17.

20. Hamid O, Robert C, Daud A, Hodi FS, Hwu WJ, Kefford R, et al. Five-year survival outcomes for patients with advanced melanoma treated with pembrolizumab in KEYNOTE-001. Ann Oncol. 2019 Apr;30(4):582–8.

21. Garon EB, Hellmann MD, Rizvi NA, Carcereny E, Leighl NB, Ahn MJ, et al. Five-Year Overall Survival for Patients With Advanced Non‒Small-Cell Lung Cancer Treated With Pembrolizumab: Results From the Phase I KEYNOTE-001 Study. J Clin Oncol. 2019 Oct 1;37(28):2518–27.

22. Peters S, Camidge DR, Shaw AT, Gadgeel S, Ahn JS, Kim DW, et al. Alectinib versus Crizotinib in Untreated *ALK* -Positive Non–Small-Cell Lung Cancer. N Engl J Med. 2017 Aug 31;377(9):829–38.

23. Goodman DT. Cemiplimab and Cutaneous Squamous Cell Carcinoma: From Bench to Bedside. JPRAS Open. 2022 Sep;33:155–60.

24. Frost N, Reck M. Non–Small Cell Lung Cancer Metastatic Without Oncogenic Alterations. Am Soc Clin Oncol Educ Book. 2024 Jun;44(3):e432524.

25. Özgüroğlu M, Kilickap S, Sezer A, Gümüş M, Bondarenko I, Gogishvili M, et al. First-line cemiplimab monotherapy and continued cemiplimab beyond progression plus chemotherapy for advanced non-small-cell lung cancer with PD-L1 50% or more (EMPOWER-Lung 1): 35-month follow-up from a mutlicentre, open-label, randomised, phase 3 trial. Lancet Oncol. 2023 Sep;24(9):989–1001.

26. Amrane K, Geier M, Corre R, Léna H, Léveiller G, Gadby F, et al. First-line pembrolizumab for non-small cell lung cancer patients with PD-L1 ≥50% in a multicenter real-life cohort: The PEMBREIZH study. Cancer Med. 2020 Apr;9(7):2309–16.

27. Sequist LV, Soria JC, Goldman JW, Wakelee HA, Gadgeel SM, Varga A, et al. Rociletinib in *EGFR* -Mutated Non–Small-Cell Lung Cancer. N Engl J Med. 2015 Apr 30;372(18):1700–9.
